# Clinical correlates of insomnia in patients with persistent post-traumatic headache compared with migraine

**DOI:** 10.1186/s10194-020-01103-8

**Published:** 2020-04-15

**Authors:** Soo-Kyoung Kim, Catherine D. Chong, Gina Dumkrieger, Katherine Ross, Visar Berisha, Todd J. Schwedt

**Affiliations:** 1 Department of Neurology and Institute of Health Science, Gyeongsang National University College of Medicine, Jinju, South Korea; 2 Department of Neurology, Mayo Clinic, 5777 East Mayo Boulevard, Phoenix, AZ 85054 USA; 3 Phoenix VA Health Care System, Phoenix, AZ USA; 4 Arizona State University, Tempe, AZ USA

**Keywords:** Posttraumatic headache, Migraine, Traumatic brain injury, Insomnia, Sleep

## Abstract

**Background:**

Close associations between insomnia with other clinical factors have been identified in migraine, but there have been few studies investigating associations between insomnia and clinical factors in patients with persistent post-traumatic headache (PPTH). The study objective was to contrast the severity of insomnia symptoms in PPTH, migraine, and healthy controls, and to identify factors associated with insomnia in patients with PPTH vs. migraine.

**Methods:**

In this cross-sectional cohort study, 57 individuals with PPTH attributed to mild traumatic brain injury, 39 with migraine, and 39 healthy controls were included. Participants completed a detailed headache characteristics questionnaire, the Migraine Disability Assessment Scale (MIDAS), Insomnia Severity Index (ISI), Hyperacusis Questionnaire (HQ), Allodynia Symptom Checklist, Photosensitivity Assessment Questionnaire, Beck Depression Inventory (BDI), State-Trait Anxiety Inventory, Post-Traumatic Stress Disorder (PTSD) checklist, Ray Auditory Verbal Learning Test, and the Trail Making Test A and B to assess headache characteristics, disability, insomnia symptoms, sensory hypersensitivities, and neuropsychological factors. Fisher’s test and one-way ANOVA or Tukey’s Honest Significant Difference were used to assess group differences of categorical and continuous data. Stepwise linear regression analyses were conducted to identify clinical variables associated with insomnia symptoms.

**Results:**

Those with PPTH had significantly higher ISI scores (16.7 ± 6.6) compared to migraine patients (11.3 ± 6.4) and healthy controls (4.1 ± 4.8) (*p* <  0.001). For those with PPTH, insomnia severity was most strongly correlated with the BDI (Spearman’s rho (*ρ*) = 0.634, *p* <  0.01), followed by Trait Anxiety (*ρ* = 0.522, *p* <  0.01), PTSD (*ρ* = 0.505, *p* <  0.01), HQ (*ρ* = 0.469, *p* <  0.01), State Anxiety (*ρ* = 0.437, *p* <  0.01), and MIDAS scores (*ρ* = 0.364, *p* <  0.01). According to linear regression models, BDI, headache intensity, and hyperacusis scores were significantly positively associated with insomnia severity in those with PPTH, while only delayed memory recall was negatively associated with insomnia severity in those with migraine.

**Conclusions:**

Insomnia symptoms were more severe in those with PPTH compared to migraine and healthy control cohorts. Depression, headache intensity, and hyperacusis were associated with insomnia in individuals with PPTH. Future studies should determine the bidirectional impact of treating insomnia and its associated symptoms.

## Background

Headache is one of the most prevalent symptoms following mild traumatic brain injury (mTBI) [1, 2]. Post-traumatic headache (PTH) as currently defined by the International Classification of Headache Disorders, third edition (ICHD-3), is a secondary headache disorder that develops within 7 days after a head injury, regaining consciousness following a head injury, or discontinuation of medications that impair ability to feel or report a headache following a head injury [3]. PTH of more than 3 months’ duration is classified as persistent PTH (PPTH) [3]. PTH is the most common type of pain attributed to mTBI, and more than one third of individuals with acute PTH have headache persistence and thus develop PPTH [1, 2, 4–6].

Individuals with acute PTH and PPTH often have a constellation of physical, psychological, and cognitive post-TBI symptoms. Previous studies have shown that insomnia and other sleep disturbances result in a delayed PTH recovery [7, 8]. Sleep is often disrupted following mTBI [9, 10] and may cause or intensify post-TBI symptoms such as depression, anxiety, suicidality, irritability, fatigue, cognitive deficits, and pain [11–15].

Insomnia is also common in patients with migraine. The close association between insomnia and migraine has been reported in several studies [16–19]. Poor sleep quality among patients with migraine is significantly associated with headache frequency and headache-related disability [20]. Since PPTH is often phenotypically very similar to migraine [2, 21, 22], we hypothesized that insomnia would associate with other symptoms similarly in those with PPTH vs. migraine [23–25].

The objective of this study was to compare the severity of insomnia symptoms in those with PPTH to migraine and healthy controls, and to identify the clinical factors associated with insomnia in patients with PPTH vs those with migraine.

## Methods

This was a cross-sectional cohort study in which participants were prospectively enrolled from November 2015 until February 2018. The data reported within this manuscript were collected as part of a United States Department of Defense funded study that aimed to compare clinical characteristics and brain imaging findings amongst those with PPTH vs. migraine [25–27] .

### Subjects

All study participants were between 18 and 65 years of age. Patients with migraine were enrolled from the Mayo Clinic Headache Center or were recruited via advertisements posted at the Mayo Clinic in Phoenix, Arizona. Patients with PPTH were enrolled from the Mayo Clinic Department of Neurology and from the Phoenix VA Health Care System. Healthy controls were recruited from the Phoenix area via advertisements and word-of-mouth. The Ohio State University TBI Identification Method was used to assess for a history of TBI [28]. Patients who had PTH attributed to mTBI for at least 3 months were included in the PPTH group regardless of their mechanism of brain injury. Headache diagnoses were made by a certified headache specialist (TS) using ICHD-3 beta diagnostic criteria [29]. Patients with PPTH were excluded if they had a personal history of moderate or severe TBI, migraine, or any other headache type prior to their injury (except infrequent episodic tension-type headaches). Those with migraine were excluded if they had a personal history of TBI. Healthy control subjects had no history of any headache type (except infrequent episodic tension-type headaches) and no history of TBI.

This study was reviewed and approved by the Mayo Clinic Institutional Review Board, the Phoenix VA Health Care System Institutional Review Board, and the US Department of Defense Human Research Protection Office.

All participants provided written informed consent prior to study participation.

### Sleep questionnaire

Symptoms of insomnia were assessed using the Insomnia Severity Index (ISI) questionnaire [30]. The ISI is a 7-item self-report questionnaire that evaluates sleep onset, sleep maintenance, early morning awakening, sleep dissatisfaction, noticeability of sleep problems by others, degree of distress, and impact of sleep disturbance on daily functioning. Each of the ISI items is rated on a scale of 0–4; total ISI scores range between 0 to 28 with higher scores indicating greater insomnia severity. The ISI total raw score is interpreted as follows: 0–7 = absence of insomnia; 8–14 = sub-threshold insomnia; 15–21 = clinical insomnia with moderate severity; 22–28 = clinical insomnia with severe severity [30–32]. Individuals answer the ISI questions based on their symptoms during the last 2 weeks.

### Headache questionnaires

Those with migraine and those with PPTH provided detailed information about their headaches including age at onset, headache duration, headache frequency, presence of aura, headache characteristics, localization, average headache intensity, associated features, comorbidity, and medical and family history, using a structured interview. Participants completed the Migraine Disability Assessment Scale (MIDAS) to assess headache-related disability during the prior 90 days [33].

### Hypersensitivity questionnaires

To investigate sensory hypersensitivities, all subjects completed the following: 1) Hyperacusis Questionnaire (HQ) which consists of 14 items designed to assess auditory symptoms and sound sensitivity. Items are rated on a 4-point scale (scores 0–3), with higher scores indicating greater sensitivity (ranging from 0 to 42) [34]. 2) Allodynia Symptom Checklist (ASC) is a 12-item questionnaire that assesses for symptoms of cutaneous allodynia during headaches. A total ASC score ≥ 3 suggests the presence of cutaneous allodynia, with higher scores indicating greater severity [35, 36]. 3) Photosensitivity Assessment Questionnaire (PAQ) which consists of 16 questions, eight of which assess for avoidance of light (photophobia) and 8 of which assess for light-seeking behavior (photophilia). Only the eight items related to photophobia were included to determine the severity of the photophobia (ranging from 0 to 8) [37, 38].

### Mood-related questionnaires

Mood-related questionnaires included the Beck Depression Inventory (BDI) for assessing depression [39], the State-Trait Anxiety Inventory for assessing anxiety [40], and the Post-traumatic Stress Disorder (PTSD) checklist in primary care (PC-PTSD) for assessing PTSD [41]. The BDI includes 21 items, with higher total scores indicating greater symptoms of depression [39]. The State-Trait Anxiety Inventory consists of 20 questions that assess how the individual feels “right now” and 20 questions assessing how the individual “generally feels”. Higher total scores suggest greater levels of anxiety [40]. The PC-PTSD is a 4-item screen for PTSD, with a total score ≥ 3 suggesting the presence of PTSD [41].

### Cognitive function evaluation

The Trail Making Test (TMT, A and B) [42] and The Rey Auditory Verbal Learning Test (RAVLT) [43] were used to evaluate cognitive function. The TMT is a test of executive function, speed of processing, and attention [44]. The RAVLT is an assessment of auditory verbal learning and memory. A list of 15 words is read aloud to the participant, and then the participant is immediately asked to recall as many words as they remember [43]. After 30-min of interpolated testing, the participant is again asked to recall the words from the first list (delayed recall) [45]. For the TMT and RAVLT, each participant’s test score was converted to a z-score based on age-matched normative data [42, 43, 45, 46].

### Statistical analyses

Descriptive statistics were used to compare demographic data and headache characteristics among groups. Frequencies were used for description of categorical variables. Fisher’s exact test was used to assess group differences for categorical data. For the comparisons of parametric data of two groups, Student’s t-test was used for parametric variables. As standard diagnostic plots did not show violation of any assumptions, non-parametric data were presented as mean ± standard deviation. For the variables applicable to only PPTH and migraine groups, such as years with headache, one-way ANOVA was used to assess the difference in mean values between groups. Tukey’s Honest Significant Difference (HSD) was used to assess mean differences among the three groups. This test performs simultaneous pairwise comparisons and controls family-wise error rate for multiple testing. According to Tukey’s HSD, age was not significantly different across the three groups. Thus, age was not corrected for in subsequent analyses. Spearman’s rank correlations between independent variables and total ISI scores were calculated to assess the relationship between insomnia severity and clinical factors. A stepwise multiple linear regression analysis was conducted to identify clinical variables associated with insomnia symptoms. The total ISI score was the dependent variable, while the candidate independent variables included sex, headache characteristics, sensory hypersensitivity scores, scores on psychological questionnaires, and cognitive test results. Patients with full datasets were included in this analysis. Statistical significance was set at *p*-value < 0.05, and 95% confidence intervals (CI) were reported as appropriate. All analyses were performed with SPSS 25.0 (Chicago, IL, USA) software.

## Results

### Subjects

A total of 137 subjects were recruited, consisting of 59 subjects with PTTH, 39 subjects with migraine, and 39 healthy controls. Two PPTH subjects were excluded due to incomplete questionnaires, leaving 57 PPTH subjects in this analysis. Of those with PPTH, 25 had mTBI due to blasts, 17 due to falls, 8 were sports-related, and 7 were caused by motor vehicle accidents. Thirteen subjects with PPTH had one lifetime mTBI, 33 had 2–5, and 11 had more than five mTBIs. Amongst those with PPTH, the clinical headache phenotypes were most similar with migraine for 41, probable migraine for 12, and tension-type headache for 4 according to the ICHD-3 criteria. Demographic profiles and clinical characteristics are shown in Table 1. Average age of subjects was 39.1 ± 10.6, and 55.6% were male. Comparing the patients with PPTH to those with migraine and healthy controls, there were no significant differences in age (mean age: PPTH = 38.2 ± 10.7 years vs. migraine = 41.3 ± 11.5 years vs. healthy control = 38.3 ± 9.5; *p =* 0.313) or sex (PPTH = males 37/57 (64.9%) vs. migraine = 16/39 (41.0%) vs. healthy control = 22/39 (56.4%), *p* = 0.072) Pairwise comparisons did show differences in sex between PPTH and migraine (*p* = 0.024) but not between PPTH and healthy control (*p* = 0.522) or between migraine and healthy control (*p* = 0.257).

**Table 1:** Subject demographics and clinical characteristics by group

	PPTH (***n*** = 57)	Migraine (***n*** = 39)	HC (***n*** = 39)	Migraine vs. PPTH ***p-***value	PPTH vs. HC ***p-***value	Migraine vs. HC ***p-***value
Age, mean (SD)	38.2 (10.7)	41.3(11.5)	38.3 (9.5)	0.329	0.997	0.433
Sex (female/male)	20/37	23/16	17/22	0.024*	0.522	0.257
Headache intensity, mean (SD)	6.0 (1.7)	6.0 (1.5)	N/A	0.981	N/A	N/A
Headache duration, hours, mean (SD)	26.3 (26.4)	27.6 (22.6)	N/A	0.805	N/A	N/A
Headache frequency, days/month, mean (SD)	16.0 (8.3)	20.5 (6.3)	N/A	0.005*	N/A	N/A
Years with headache, mean (SD)	11.0 (8.8)	24.9 (14.6)	N/A	< 0.001*	N/A	N/A
MIDAS, mean (SD)	64.2 (57.4)	52.2 (37.6)	N/A	0.256	N/A	N/A
ISI, mean (SD)	16.7 (6.6)	11.3 (6.4)	4.1 (4.8)	< 0.001*	< 0.001*	< 0.001*
HQ, mean (SD)	22.7 (10.6)	14.5 (9.1)	5.7 (4.8)	< 0.001*	< 0.001*	< 0.001*
Allodynia, n (%)	38 (66.7%)	26 (66.7%)	N/A	1.00	N/A	N/A
PAQ, mean (SD)	4.6 (2.6)	3.8 (2.5)	1.1 (1.5)	0.270	< 0.001*	< 0.001*
BDI, mean (SD)	17.8 (9.4)	8.7 (6.0)	2.1 (3.6)	< 0.001*	< 0.001*	< 0.001*
State, mean (SD)	38.8 (13.2)	33.4 (9.1)	23.6 (5.7)	0.038*	< 0.001*	< 0.001*
Trait, mean (SD)	44.6 (14.1)	39.4 (10.9)	26.5 (6.9)	0.079	< 0.001*	< 0.001*
PTSD, n (%)	39 (68.4%)	2 (5.1%)	2 (5.1%)	< 0.001*	< 0.001*	1.00

*SD* Standard deviation, *N/A* Not applicable, *PPTH* Persistent post-traumatic headache, *HC* Healthy controls, *Headache duration* Average hours of headache per attack; *Headache intensity* Average pain intensity of headache per attack, *Headache Frequency* Number of headache days per month, *Years with headache* Years lived with headache, *MIDAS* Migraine Disability Assessment Scale, *ISI* Insomnia Severity Index, *ASC* Allodynia Symptom Checklist, *HQ* Hyperacusis Questionnaire, *PAQ* Photosensitivity Assessment Questionnaire, *BDI* Beck Depression Inventory, *State* State anxiety score, *Trait* Trait anxiety score, *PTSD* The primary care Post-traumatic Stress Disorder screen

(Statistical threshold used = * *p* < 0.05)

The PPTH group had a lower headache frequency than the migraine group (mean headache days/30-day month = 16.0 ± 8.3 vs. 20.5 ± 6.3, *p =* 0.005) and fewer mean years with headache (11.0 ± 8.8 years vs. 24.9 ± 14.6 years, *p <* 0.001). There were not significant differences in mean total MIDAS scores (64.2 ± 57.4 in the PTTH group vs. 52.2 ± 37.6 in the migraine group, (*p =* 0.256) (Table 1).

### Insomnia, hypersensitivity and mood related scores

**Fig. 1:**
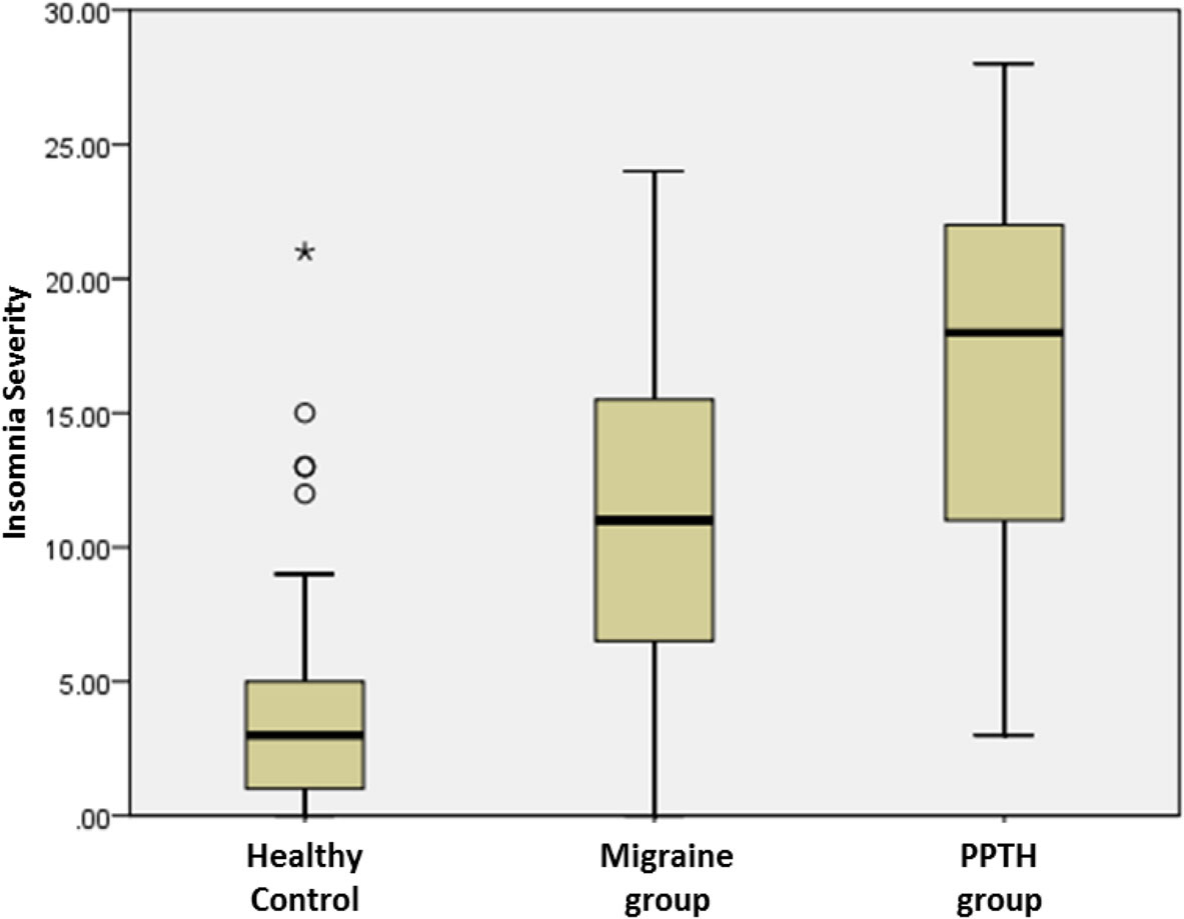
Insomnia Severity Index scores among groups. PPTH: Persistent posttraumatic headache

**Fig. 2:**
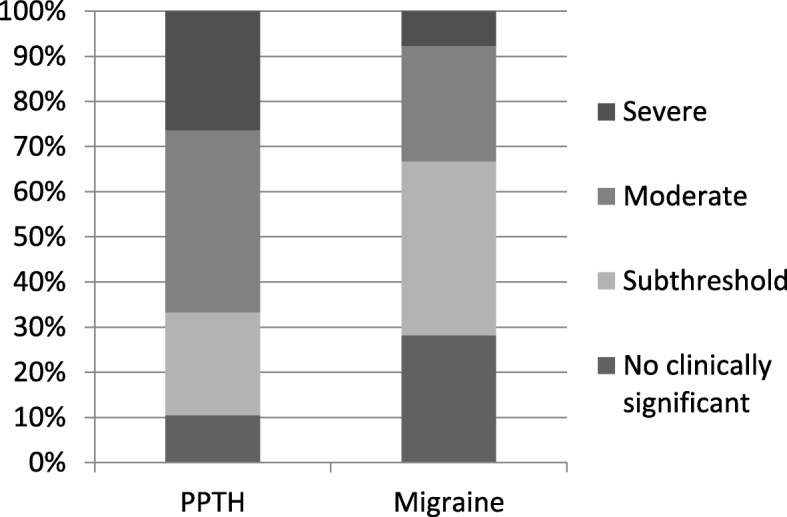
Distribution of insomnia severity in subjects with persistent post-traumatic headache and migraine. PPTH: Persistent posttraumatic headache

The mean ISI scores were significantly higher in the PPTH group compared with migraine and healthy control groups (16.7 ± 6.6 vs 11.3 ± 6.4 and 4.1 ± 4.8, *p* <  0.001) (Fig. 1). The proportion of individuals with severe insomnia with a total ISI score = 22–28 (26.3% vs 7.7%, *p* <  0.001) and moderate insomnia with a total ISI score = 15–21 (40.4% vs 25.6%, *p* <  0.001) was significantly higher in the patients with PPTH than those with migraine (Fig. 2). The mean total score on the HQ (22.7 ± 10.6 vs 14.5 ± 9.1 and 5.7 ± 4.8, *p* <  0.001) was higher in the PPTH group compared to the migraine and healthy control group. Based on ASC scores ≥3, 66.7% (38/57) of patients with PPTH and 66.7% (26/39) of those with migraine had cutaneous allodynia (*p =* 1). The mean total PAQ score of photophobia was not different in the PPTH group compared to the migraine group (4.6 ± 2.6 vs 3.8 ± 2.5, *p* = 0.270).

The mean total BDI scores (PPTH: 17.8 ± 9.4 vs migraine 8.7 ± 6.0 and healthy control 2.1 ± 3.6, *p* <  0.001) and state anxiety scores (38.8 ± 13.2 vs 33.4 ± 9.1 and 23.6 ± 5.7, *p = 0.038* and *p <* 0.001) were significantly higher in the PPTH group compared to the migraine and healthy control groups. Trait anxiety scores (44.6 ± 14.1 vs 39.4 ± 10.9 and 26.5 ± 7.4, *p = 0.079* and *p* <  0.001) were significantly higher in the PPTH group compared to the healthy control group, but were not significantly higher in the PPTH group compared to the migraine group. Based on scores ≥3 for the PTSD, 68.4% (39/57) of patients with PPTH registered as having PTSD compared to 5.1% (2/39) of those with migraine (*p <* 0.001) (Table 1).

### Cognitive evaluation

The results of cognitive tests are presented in Table 2. Z-scores were calculated for scores from each group using normative values. The TMT A mean raw score was 27.6 ± 9.4 (z-score 0.18) in the PPTH group vs 27.7 ± 11.0 (z-score 0.13) in the migraine group (*p* = 0.973) and 23.1 ± 5.1 (z-score 0.59) for healthy controls (*p* = 0.126). The TMT B mean raw score was 69.5 ± 26.7 (z-score 0.16) in the PPTH group vs 65.7 ± 29.6 (z-score 0.35) in the migraine group (*p* = 0.585) and 58.1 ± 21.1(z-score 0.52) for healthy controls (*p* = 0.153). Time to completion did not significantly differ by the groups for either TMT A or TMT B. The z-scores with mean raw immediate recall scores (PPTH: − 1.22 (5.5 ± 2.1) vs migraine: − 0.67 (6.3 ± 1.6), *p* = 0.087, and healthy control: − 0.69 (6.4 ± 1.4), *p* = 0.109) and delayed recall scores (− 1.01 (8.9 ± 4.2) vs − 0.32 (10.2 ± 3.0), *p* = 0.065 and − 0.34 (10.4 ± 2.9), *p* = 0.075) of RAVLT in the PTTH group were not significantly different than those of the migraine group and healthy controls.

**Table 2:** Mean raw and z-scores for cognitive tests

	PPTH (*n* = 57)		Migraine (*n* = 39)		HC (*n* = 39)		Migraine vs. PPTH *p-*value	PPTH vs. HC *p-*value	Migraine vs. HC *p-*value
	Mean (SD)	z-score	Mean (SD)	z-score	Mean (SD)	z-score			
TMT A	27.6 (9.4)	0.18	27.7 (11.0)	0.13	23.1 (5.1)	0.59	0.973	0.126	0.115
TMT B	69.5 (26.7)	0.16	65.7 (29.6)	0.35	58.1(21.1)	0.52	0.585	0.153	0.702
Immediate recall	5.5 (2.1)	−1.22	6.3 (1.6)	−0.67	6.4 (1.4)	−0.69	0.087	0.109	0.995
Delayed recall	8.9 (4.2)	−1.01	10.2 (3.0)	−0.32	10.4 (2.9)	−0.34	0.065	0.075	0.998

Results of Tukey HSD performed on z-scores. *SD* Standard deviation, *PPTH* Persistent post-traumatic headache, *HC* Healthy controls, *TMT* Trail making test

### Clinical factors associated with insomnia

Among the PPTH group, BDI scores exhibited the strongest correlation with insomnia severity scores (Spearman’s rho (*ρ*) = 0.634, *p* <  0.01), followed by trait anxiety scores (*ρ* = 0.522, *p <* 0.01), PTSD scores (*ρ* = 0.505, *p <* 0.01), HQ scores (*ρ* = 0.469, *p <* 0.01), state anxiety scores (*ρ* = 0.437, *p <* 0.01), MIDAS scores (*ρ* = 0.364, *p <* 0.01) and ASC scores (*ρ* = 0.284, *p* <  0.05). In the PPTH group, there were no significant associations between insomnia severity and cognitive function according to TMT A and B and immediate and delayed recall of the RAVLT. However, the migraine group showed a significant correlation between increased insomnia severity and poorer performance for delayed recall on the RAVLT (*ρ* = − 0.390, *p* <  0.05) (Table 3).

**Table 3:** Spearman correlation coefficients between Insomnia Severity Index and clinical parameters

		PPTH (*n* = 57)	Migraine (*n* = 39)
Headache	Headache intensity	0.235	− 0.073
	Headache duration	− 0.116	− 0.190
	Headache frequency	0.069	0.045
	Years with headache	−0.009	− 0.124
	MIDAS	0.364^b^	0.268
Hypersensitivity	HQ	0.469^b^	0.240
	ASC	0.284^a^	0.197
	PAQ	0.181	−0.057
Mood	BDI	0.634^b^	0.308
	PTSD	0.505^b^	0.097
	State	0.437^b^	0.046
	Trait	0.522^b^	0.156
Cognitive function	TMT A	−0.132	−0.041
	TMT B	−0.154	−0.071
	Immediate recall	−0.149	−0.180
	Delayed recall	−0.164	−0.390^a^

*PPTH* Persistent Post traumatic headache, *MIDAS* Migraine Disability Assessment Scale, *HQ* Hyperacusis Questionnaire, *ASC* Allodynia Symptom Checklist, *PAQ* Photosensitivity Assessment Questionnaire, *BDI* Beck Depression Inventory, *PTSD* Post-traumatic Stress Disorder, *State* State anxiety score, *Trait* Trait anxiety score, *TMT* Trail making test

^a^Correlation is significant at the 0.05 level (2-tailed)

^b^Correlation is significant at the 0.01 level (2-tailed)

In the migraine group, the stepwise multiple regression analysis produced a model including one variable that explained 12.9% of insomnia severity variance. The delayed memory recall was the only parameter significantly associated with insomnia severity (β = − 0.359, *p =* 0.025) in the migraine group. In the PPTH group, regression analyses produced a model with 3 variables explaining 51.7% of insomnia severity variance (R^2^ = 0.517; adjusted R^2^ = 0.489*, F = 56.11*). The total BDI score (β = 0.555, *p <* 0.001) was most strongly associated with insomnia severity, followed by headache intensity (β = 0.230, *p =* 0.019), and HQ score (β = 0.220, *p =* 0.047) (Table 4).

**Table 4:** Multiple stepwise linear regression analysis for association of insomnia severity scores

		Unstandardized Coefficients		Standardized Coefficients	t	*p*	*R*^*2*^	*Adjusted R*^*2*^
		B	SE	β				
Migraine	(Constant)	10.652	1.01		10.548	0	0.129	0.106
	**Delayed recall**	−1.996	0.852	−0.359	−2.343	0.025*		
PPTH	(Constant)	1.219	2.810		0.434	0.666	0.517	0.489
	**BDI**	0.390	0.076	0.555	5.133	< 0.001**		
	**Headache intensity**	0.906	0.376	0.230	2.411	0.019*		
	**HQ**	0.137	0.067	0.220	2.034	0.047*		

Dependent Variable: Insomnia Severity Index score (* *p <* 0.05, **p < 0.01)

Independent variables such as sex, headache pain intensity, headache duration, headache frequency, years with headache, MIDAS, HQ, ASC, PAQ, BDI, PTSD, State anxiety score, Trait anxiety score, TMT A and B, Immediate recall, and Delayed recall were included as candidate variables

*PPTH* Persistent Post traumatic headache, *MIDAS* Migraine Disability Assessment Scale, *HQ* Hyperacusis Questionnaire, *ASC* Allodynia Symptom Checklist, *PAQ* Photosensitivity Assessment Questionnaire, *BDI* Beck Depression Inventory, *PTSD* Post-traumatic Stress Disorder, *TMT* Trail making test

## Discussion

In this study, we found that PPTH and migraine patients had more severe insomnia symptoms relative to a healthy control cohort. The severity of insomnia symptoms was greater in individuals with PPTH attributed to mTBI compared to those with migraine. In those with PPTH, depression, headache intensity, and hyperacusis were the strongest factors associated with insomnia severity. Amongst those with migraine, delayed memory recall was related to insomnia severity. Our finding suggests that insomnia is prevalent and more severe in PPTH patients than migraine patients; nevertheless the majority of PPTH patients in this study had migraine-like headaches. These results indicate that we need to assess for insomnia in patients with PPTH, a condition that could be easily overlooked if the clinician focuses attention entirely on the headache and associated symptoms. Although the directionality of the relationships between insomnia and these associated symptoms cannot be determined from this cross-sectional study, it is possible that effective management of insomnia could relieve some of these associated symptoms and vice-versa. Different than PPTH, our study suggests that insomnia might affect delayed memory recall in patients with migraine. Therefore, insomnia might have different clinical implications in those with PPTH vs. migraine, and these implications should be properly considered according to the underlying headache type [47].

Headache and insomnia frequently co-occur, and are mutually interacting conditions following TBI. Prior studies demonstrated that 30 ~ 70% of patients with TBI complained of sleep disturbance [48, 49], and that insomnia was a potential risk factor for PTH [7]. Insomnia and other sleep disturbances resulted in a delayed PTH recovery and could lead to PPTH [7, 8]. Mechanistically, it is possible that insomnia could affect the descending pain inhibitory control system and enhance pain perception in patients with PTH [50, 51]. In fact, in our study there was a positive correlation between insomnia severity with headache intensity and with symptoms of cutaneous allodynia. A recent study proposed that the perivascular glymphatic exchange impairment caused by TBI and sleep disruption may impair the clearance of neuropeptides such as calcitonin gene-related peptide (CGRP) involved in the pathogenesis of PTH [11]. However, there is still a lack of studies investigating mechanisms linking the persistence of PTH and sleep disturbance attributed to TBI. In our study, the high prevalence (66.3%) and severity of insomnia in those with PPTH suggest that insomnia might be associated with the persistence of pain in PTH after mTBI.

The pain of PPTH could also contribute to and exacerbate insomnia. Although markers of headache burden, such as years with headache and headache frequency, were lower in the PPTH group compared to the migraine group, insomnia was more prevalent and more severe among those with PPTH. It is possible that those with more years of headache adapt to their headache symptoms, resulting in less impact on sleep over time. These results could also suggest that factors in addition to headache may be involved in insomnia of PPTH and migraine.

In our study, depression was the strongest predictor of insomnia in those with PPTH. Our results also demonstrated that mood alterations including depression, anxiety and PTSD symptoms were reported more frequently in those with PPTH than those with migraine and healthy controls. Depression, anxiety, PTSD and suicidality symptoms are common following mTBI [7, 52]. Those with PPTH following mTBI might have low serotonin levels, contributing to development of psychological symptoms and insomnia [53–56]. In addition, headache and non-headache pain are common comorbid conditions in those with PPTH, contributing to sleep disturbances and mood alterations [57]. Our study showed that insomnia is associated with depression and headache intensity in those with PPTH, though the cause-effect relationship is unclear.

Hyperacusis was significantly associated with insomnia in those with PPTH in our data. Hyperacusis is the condition of reduced tolerance or increased sensitivity to sound, and is characterized by excessive loudness, troublesomeness, anxiety, and pain when the person is exposed to sound [58, 59]. Auditory symptoms including subjective hearing loss, tinnitus, and hyperacusis are common in those with mTBI, and 67.3% of non-blast mTBI patients reported hyperacusis according to a recent patient-reported outcome measures study [60]. Mechanisms underlying hyperacusis following mTBI are unknown, although proposed theories suggest a tonotopic reorganization and hyperexcitability of the auditory cortex involving a malfunction of 5-HT [61], a release of endogenous opioid peptides [62], and a decreased GABA_A_-mediated inhibition in the inferior colliculus [63]. Many studies have shown that the sleep disturbance generated by hyperacusis is mediated by psychological factors [58, 64, 65]. Anxiety and hyperresponsiveness to every sound while one is trying to sleep could result in insomnia. Fortunately, a recent study demonstrated that prophylactic treatment of migraine is associated with improvements in hyperacusis, suggesting that hyperacusis might share a pathophysiologic basis with migraine [66]. It is yet to be conclusively demonstrated whether effective treatment of PPTH is associated with reductions in hyperacusis.

In this study, one third of individuals with migraine reported symptoms of insomnia, though the sample size was small. Prior studies have demonstrated that individuals with migraine are at an increased risk of having insomnia, and individuals with insomnia have a higher risk of migraine compared to individuals without insomnia [17, 18, 67, 68]. In our study, insomnia severity was associated with delayed memory recall in individuals with migraine. The close relationship between insomnia and cognitive dysfunction has been described in previous studies [69–71]. Future studies with larger sample sizes are needed to further determine the effects of insomnia on memory impairment in individuals with migraine.

This is the first study to compare the severity of insomnia symptoms in individuals with PPTH vs. those with migraine. The identification of associations between symptoms of depression, hyperacusis, and headache intensity with symptoms of insomnia in those with PPTH yields relational insights into these common post-TBI symptoms. However, there are several limitations in our study. First, the cross-sectional design does not allow for determining the direction of relationships between insomnia and its associated symptoms. Second, in order to avoid issues with recall bias, the response period for the insomnia questionnaire was limited to the prior 2 weeks. Although it is likely that symptoms during that 2 week period are reflective of symptoms over a longer time-period, this cannot be confirmed. It is not known if the individuals in this study had insomnia prior to development of PPTH or migraine, or if insomnia developed after headache onset. Third, we didn’t have objective sleep measures and information about other sleep related disturbance (e.g., obstructive sleep apnea, restless leg syndrome). Fourth, we did not evaluate additional auditory symptoms other than hyperacusis (e.g. tinnitus, hearing loss) and did not perform objective audiological investigations to identify or quantify them. Fifth, since many patients in our study were recruited from a specialty headache clinic, it is not clear if the results would be generalizable to the general population of people with PPTH.

## Conclusion

Although the vast majority of individuals with PPTH in this study had migraine-like headaches, insomnia was more severe among individuals with PPTH compared to those with migraine. Among those with PPTH, there were associations between insomnia symptom severity with symptoms of depression, headache intensity, and hyperacusis, relationships not shared by those with migraine. The frequency and severity of insomnia symptoms found in this study suggest that the clinician should assess patients with PPTH for insomnia. Although not conclusive from this cross-sectional study, the associations between insomnia with depression, headache intensity, and hyperacusis at least suggest that treatment of insomnia could lead to improvements in other symptoms associated with PPTH. Future studies should determine the bidirectional impact of treating insomnia and its associated symptoms.

## Data Availability

The dataset supporting the conclusions of this article is available via the Federal Interagency Traumatic Brain Injury Research (FITBIR) informatics system.

## Abbreviations

mTBI: mild traumatic brain injury
PTH: Post-traumatic headache
ICHD-3: The International Classification of Headache Disorders, third edition
PPTH: Persistent post-traumatic headache
HC: Healthy controls
MIDAS: Migraine Disability Assessment Scale
ISI: Insomnia Severity Index
ASC: Allodynia Symptom Checklist
HQ: Hyperacusis Questionnaire
PAQ: Photosensitivity Assessment Questionnaire
BDI: Beck Depression Inventory
STATE: State Anxiety Inventory
PTSD: Post-traumatic Stress Disorder
PC-PTSD: The primary care Post-traumatic Stress Disorder
TMT: Trail Making Test
RAVLT: Rey Auditory Verbal Learning Test
SD: Standard deviation
N/A: Not applicable
ANOVA: Analysis of variance
